# Self-Assembling Scaffolds Supported Long-Term Growth of Human Primed Embryonic Stem Cells and Upregulated Core and Naïve Pluripotent Markers

**DOI:** 10.3390/cells8121650

**Published:** 2019-12-16

**Authors:** Christina McKee, Christina Brown, G. Rasul Chaudhry

**Affiliations:** 1 Department of Biological Sciences, Oakland University, Rochester, MI 48309, USA; cmmckee@oakland.edu (C.M.); brown3@oakland.edu (C.B.); 2 OU-WB Institute for Stem Cell and Regenerative Medicine, Rochester, MI 48309, USA

**Keywords:** embryonic stem cells, three-dimensional, self-assembling scaffold, pluripotency, culture conditions, expansion, growth, niche

## Abstract

The maintenance and expansion of human embryonic stem cells (ESCs) in two-dimensional (2-D) culture is technically challenging, requiring routine manipulation and passaging. We developed three-dimensional (3-D) scaffolds to mimic the in vivo microenvironment for stem cell proliferation. The scaffolds were made of two 8-arm polyethylene glycol (PEG) polymers functionalized with thiol (PEG-8-SH) and acrylate (PEG-8-Acr) end groups, which self-assembled via a Michael addition reaction. When primed ESCs (H9 cells) were mixed with PEG polymers, they were encapsulated and grew for an extended period, while maintaining their viability, self-renewal, and differentiation potential both in vitro and in vivo. Three-dimensional (3-D) self-assembling scaffold-grown cells displayed an upregulation of core pluripotency genes, *OCT4*, *NANOG*, and *SOX2*. In addition, the expression of primed markers decreased, while the expression of naïve markers substantially increased. Interestingly, the expression of mechanosensitive genes, *YAP* and *TAZ,* was also upregulated. YAP inhibition by Verteporfin abrogated the increased expression of *YAP*/*TAZ* as well as core and naïve pluripotent markers. Evidently, the 3-D culture conditions induced the upregulation of makers associated with a naïve state of pluripotency in the primed cells. Overall, our 3-D culture system supported the expansion of a homogenous population of ESCs and should be helpful in advancing their use for cell therapy and regenerative medicine.

## 1. Introduction

Pluripotency is defined by the potential of a cell to differentiate into germline cells as well as the cells of all three germ layers [1,2]. During development, pluripotency ranges from the formation of the epiblast until gastrulation and lineage commitment, resulting in a population of cells representing a range of pluripotent states [3,4]. Pluripotency in vitro is determined by the developmental stage from which the cell line is derived and by culture conditions [5,6,7,8] with distinct naïve and primed states of pluripotency corresponding to in vivo pre-implantation and post-implantation epiblast cells, respectively [4]. 

Embryonic stem cells (ESCs) display unlimited self-renewal and differentiation potential in vitro [9], making them an ideal source for the development of cell therapies and regenerative medicine applications. However, the clinical use of ESCs requires a high quality and quantity of cells, which is limited by currently used culturing techniques [10]. ESCs are typically grown as a monolayer in two-dimensional (2-D) plastic culture plates coated with extracellular matrix (ECM) components (such as gelatin from porcine skin [11], matrigel [12], laminin [13], fibronectin [14], vitronectin [15], or collagen [16]) or a mouse embryonic fibroblast (MEF) feeder layer to aid in attachment [17]. Monolayer culture also necessitates routine passaging and the removal of spontaneously differentiated colonies to maintain the self-renewal and potency of cells [18], which pose a major impediment to the large-scale expansion of cells. Generally, 2-D culturing methods often lead to heterogeneous cell populations as well as batch-to-batch variation. 

Moreover, 2-D culture conditions lack the intricacies necessary to mimic the ESC niche, dynamic, and specialized three-dimensional (3-D) microenvironments, which are critical for regulating cell fate in vivo. Furthermore, native 3-D niches allow for complex spatial interactions between cells ECM components as well as gradients of nutrients, oxygen, and metabolic waste [19,20,21,22,23,24]. The microenvironment is important for the self-renewal of ESCs, since cell fate and function is affected by the composition and organization of the ECM [25,26,27] as well as mechanical forces generated between cells and attachment substrates [25,28,29,30,31]. Furthermore, mechanical forces generated by the expansion of the blastocoel have been shown to play an important role in blastocyst lineage formation, stimulating the generation of pluripotent cells [32]. These early morphogenic events in the mammalian embryo indicate a significant interaction between mechanical forces, cell polarity, and the regulation of gene expression in cell fate determination [33]. We hypothesized that 3-D culture would better mimic the in vivo microenvironment, promoting the proliferation and maintenance of human ESCs. 

We have previously demonstrated that 3-D self-assembling scaffolds composed of thiolated dextran and 4-arm polyethylene glycol (PEG) functionalized with acrylate groups (Dex-SH/PEG-4-Acr) supported the growth and maintenance of naïve mouse ESCs [34]. However, these 3-D culture conditions failed to support the growth of human primed ESCs. Unlike mouse ESCs, human cells display poor viability and clonogenicity following single cell dissociation [9,10].

In this study, we describe 3-D hydrogel scaffolds that support the long-term growth and maintenance of human primed ESCs (H9). Chemically cross-linked hydrogels were formed by a Michael-type addition reaction by combining two 8-arm PEG polymers functionalized with either thiol (PEG-8-SH) or acrylate end groups (PEG-8-Acr). The PEG-8-SH/PEG-8-Acr scaffold provided a microenvironment that maintained self-renewal and pluripotency for an extended time period. Interestingly, H9 cells displayed an upregulation of core pluripotency markers during 3-D culture. However, the expression of core markers reverted to normal levels when 3-D grown cells were subcultured under 2-D culture conditions. Interestingly, 3-D cultured H9 cells also exhibited a significantly higher expression of naïve pluripotent markers when compared to human naïve ESCs (Elf1) cultured under 2-D conditions. Our results suggest the importance of the 3-D scaffold microenvironment in maintaining the stemness of ESCs. 

## 2. Materials and Methods

### 2.1. Maintenance of Human ESCs in 2-D culture

H9 cells, derived from a human blastocyst [35], obtained from WiCell (Madison, WI, USA) were maintained in the culture medium containing Knockout/F12 Dulbecco’s Modified Eagle Media (DMEM; Life Technologies, Carlsbad, CA, USA) with 20% KnockOut serum replacement (Life Technologies), 0.1 mM 2-mercaptoehtanol (Life Technologies), 1% GlutaMax (Life Technologies), and 1% non-essential amino acid solution (Life Technologies), supplemented with 20 ng/mL of basic fibroblast growth factor (FGF2, Prospec, Ness Ziona, Israel) and 10 μM ROCK inhibitor, Y-27632 (Cayman Chemical, Ann Arbor, MI, USA) and subcultured by manual passaging. For dissociation into single cells, H9 were treated with Accutase (Thermo Fisher Scientific, Waltham, MA, USA) for 5 min, and then cells were centrifuged at 200 g for 5 min. The supernatant was aspirated, and the cell pellet was resuspended in H9 culture media for plating. 

Elf1 cells, isolated from a cryopreserved 8-cell human embryo [36], were maintained in the culture medium containing KnockOut/F12 DMEM with GlutaMax (Life Technologies) with 20% KnockOut serum replacement (Life Technologies), 1 mM sodium pyruvate (Life Technologies), 0.1mM 2-mercaptoehtanol (Life Technologies), 1% non-essential amino acids (Life Technologies), and 0.2% penicillin–streptomycin solution (Life Technologies), supplemented with 12 ng/mL of FGF2 (Prospec), 1.5 μM CHIR99021 (Caymen Chemical), 0.4 μM PD03296501 (Caymen Chemical), and 0.01 μg/mL human LIF (Prospec) and cultured according to the published protocol [36]. 

### 2.2. Selection and Composition of Scaffolds for 3-D Culture of Human ESCs

Several polymers end functionalized with thiol and acrylate end groups were tested for their ability to self-assemble and form hydrogels scaffolds via a thiol–Michael addition reaction, allowing for the formation of covalent bonds by the addition of a nucleophile to a nucleophile acceptor containing an α,β-unsaturated carbonyl compound [37]. Then, the scaffolds were tested in various compositions to determine the optimal concentration, molar ratio, and degree of modification that would best support the growth of human ESCs. Our preliminary results showed that scaffolds made using 8-arm PEG polymers yielded optimal growth. These scaffolds were prepared using 8-arm PEG-thiol (PEG-8-SH, 20 kDa) and 8-arm PEG-acrylate (PEG-8-Acr, 20 kDa) purchased from JenKem Technology USA (Plano, TX, USA). Functionalized PEG polymers were stored at −20 °C and protected from light. 

**Figure 1:**
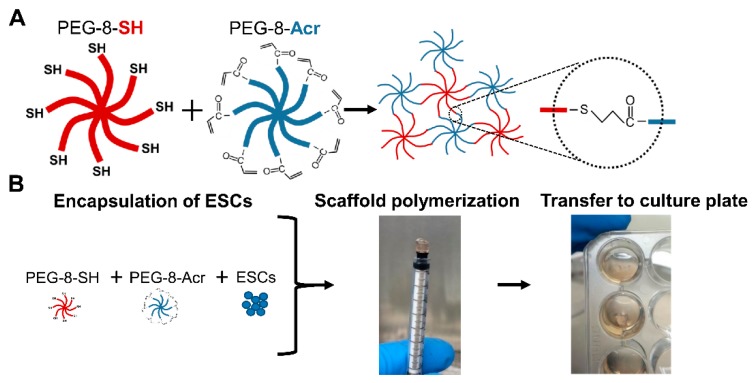
Schematic of self-assembling scaffolds. (**A**) Self-assembly of functionalized polymers, 8-arm polyethylene glycol functionalized with thiol (PEG-8-SH) and acrylate (PEG-8-Acr) via a thiol–Michael addition reaction. (**B**) The encapsulation of H9 cells human embryonic stem cells (ESCs), was achieved upon mixing with the self-assembling polymers in a syringe mold. Following polymerization, the scaffolds were then incubated in culture plates containing medium.

The preparation of scaffolds for the 3-D culture of ESCs is depicted in Figure 1. Briefly, PEG-8-SH and PEG-8-Acr polymers were dissolved at a concentration of 2.5 *w/v* % (dry weight of polymer per volume of culture medium), combined at a 1:1 molar ratio and mixed with cells. Then, the resulting mixture was transferred to a 1 cc syringe mold for polymerization. After self-assembly, scaffolds were placed in a 24-well culture plate (Fisher Scientific, Pittsburgh, PA, USA), supplemented with culture medium, and maintained in a 5% CO_2_ incubator at 37 °C. The medium was changed daily or as needed. Cell growth in the scaffolds was monitored by phase-contrast microscopy.

### 2.3. Cell Proliferation and Viability Assays

The growth rate of cells grown under 2-D and 3-D culture conditions were analyzed at various time intervals using a proliferation assay. Briefly, triplicate samples were treated with 5 mg/mL 3-(4,5-dimethylthiazol-2-yl)-2,5-diphenyltetrazolium bromide (MTT) reagent (Sigma, St. Louis, MO, USA), protected from light, and incubated at 37 °C for 4 h to obtain insoluble formazan, which was then solubilized using 15:1 isopropanol/hydrochloride. Then, the absorbance of the solubilized formazan was measured at 570 nm using an Epoch microplate reader (BioTek, Winooski, VT), and the background absorbance of the cells was subtracted from all measured values. The viability of encapsulated cells was determined by direct microscopic counts and trypan blue exclusion assay. Briefly, cells were counted using a hemocytometer and cells stained blue were considered non-viable. 

### 2.4. Differentiation of Human ESCs 

Germ layer differentiation was achieved by the spontaneous formation of embryoid bodies (EBs). ESCs were allowed to spontaneously aggregate for 3 days in non-adherent flat-bottomed 96-well plates in their respective ESC culture medium containing growth factors. Then, the resultant EBs were transferred to 0.1% gelatin-coated wells for adherent growth and grown in high-glucose DMEM supplemented with 10% fetal bovine serum (FBS). Spontaneous differentiation into all three germ layers was assessed by germ layer marker expression by quantitative real time-polymerase chain reaction (qRT-PCR) and immunocytochemistry.

### 2.5. Teratoma Assay

For teratoma formation, ESCs were harvested following accutase treatment, washed and resuspended in PBS, and mixed with an equal volume of matrigel (BD Biosciences, San Jose, CA, USA). Cells (1 × 10^6^) were subcutaneously injected (20 µL) using a Hamilton syringe into 4-week-old male immune-compromised SCID (severe combined immunodeficient) Beige mice (Fox Chase SCID Beige, Charles River, Wilmington, MA, USA). Animals were monitored daily and humanely euthanized by CO_2_ overdose after teratoma formation at 10–12 weeks. Teratomas were explanted, and teratoma tissue was either fixed for histological analysis or flash frozen in liquid nitrogen for RNA isolation. Teratoma assays were performed in triplicate. All the procedures involving animals were reviewed and approved by the Institutional Animal Care and Use Committee of Oakland University (IACUC protocol number: 17031).

### 2.6. Gene Expression Analysis

Transcriptional analysis was performed by qRT-PCR. Briefly, cells, scaffolds, and teratoma tissue (100–250 mg) were harvested and total cellular mRNA was isolated following the manufacturer’s instructions using the GeneJET RNA purification kit (Thermo Fisher Scientific) and RNeasy Midi kit (Qiagen, Germantown, MD, USA), respectively. cDNA was synthesized with the iScript kit (BioRad, Hercules, CA, USA). qRT-PCR was performed using SsoAdvanced SYBR Green Supermix (Bio-Rad) and the CFX90 Real-Time PCR system. The primers (IDT Technologies, Coralville, IA, USA) used in this study are in Table 1. All reactions were prepared in triplicate and normalized to reference genes, *HMBS*, *GAPDH*, and *β-ACTIN*. 

### 2.7. Immunocytochemical Analysis 

Protein expression was determined by immunocytochemical staining using selected antibodies. H9 cells grown under 2-D culture conditions on coverslips, H9 cells encapsulated in the self-assembling scaffolds, and harvested teratoma tissue were fixed in 4% paraformaldehyde for 10 min, 30 min, and overnight, respectively. Subsequently, teratoma tissue and scaffolds were embedded and frozen in optimal cutting temperature (O.C.T) compound and cryosectioned into 10 μm sections. For immunochemical analysis, fixed cells and cryosections were permeabilized with 0.5% Triton X-100 (Sigma) for 10 min and blocked with 2% bovine serum albumin (BSA; Sigma) for 1 h at room temperature. Next, samples were incubated with primary antibodies (1:100 dilution) overnight at 4 °C. Primary antibody-treated samples were washed three times with phosphate buffer saline (PBS), stained with secondary antibody at 1:200 dilutions for 2 h at 37 °C, and counterstained with 1 mg/mL 4′,6-diamidino-2-phenylindole (DAPI; Life Technologies). The stained samples were visualized by using confocal microscopy. The antibodies used are listed in Table 2.

### 2.8. Effect of YAP Inhibitor on Human ESCs Grown in 3-D Self-Assembling Scaffolds 

For analysis of yes-associated protein (YAP) signaling in 3-D culture, human ESCs were grown in self-assembling scaffolds for 7 days, and then treated with 2 µM YAP inhibitor (YAPi), Verteporfin (VP, R&D Systems, Minneapolis, MN), for an additional 7 days. Cell growth in the scaffolds was monitored by light microscopy. ESCs grown in 3-D self-assembling scaffolds were harvested after 14 days of culture with and without YAPi treatment as a control. Cells were harvested for RNA and immunocytochemical analysis. 

### 2.9. Statistical analysis

Data are presented as mean ± standard error of the mean (SE). One-way ANOVA analysis was performed and analyzed for unequal variances using post hoc tests for multiple comparisons. Results with a p-value less than 0.05 were considered to be significant (* *p* < 0.05 and ** *p* < 0.01). All analyses were performed using SPSS version 26 (SPSS Inc., Chicago, IL., USA).

## 3. Results

### 3.1. Growth and Characterization of H9 Cells Grown under 3-D Culture Conditions

**Figure 2:**
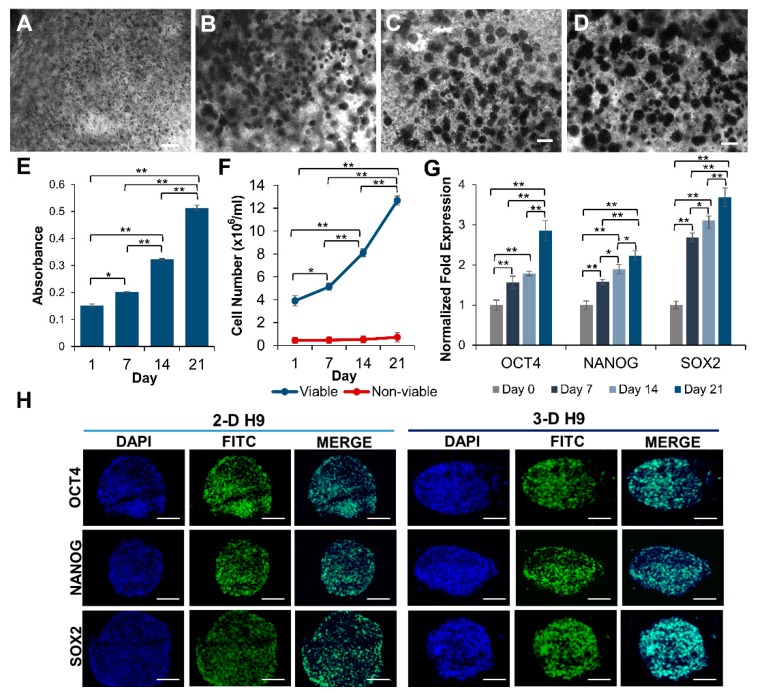
Growth of pluripotent human ESCs in 3-D self-assembling scaffolds. (**A**–**D**) Clonal growth of ESCs (H9 cells) encapsulated in PEG-8-SH/PEG-8-Acr scaffolds and incubated in culture medium was observed by light microscopy at 0, 7, 14, and 21 days. (**E**) Quantitative determination of cell proliferation by 3-(4,5-dimethylthiazol-2-yl)-2,5-diphenyltetrazolium bromide (MTT) assay using microplate reader. Results were expressed as the absorbance ± standard error (SE) with a significant increase in cell number. (**F**) Growth of ESCs encapsulated in 3-D scaffolds was assayed by direct counts using a hemocytometer, and cell viability was determined by trypan blue exclusion assay at various time intervals. Data presented as cell number (×10^6^ cells/mL) ± SE. (**G**) Expression of selected pluripotency markers, *OCT4*, *NANOG*, and *SOX2*, in ESCs cultured in self-assembling scaffolds for 0, 7, 14, and 21 days as determined by qRT-PCR. The expression of genes at day 0 was set to 1 and results were expressed as the fold expression ± SE normalized to reference genes *HMBS*, *GAPDH,* and *β-ACTIN* (* *p* < 0.05 and ** *p* < 0.01). (**H**) Confocal images (20X) of 2-D and 3-D grown ESCs displaying the expression of pluripotent proteins, OCT4, NANOG, and SOX2. All scale bars represent 100 μm.

H9 cells encapsulated in self-assembling scaffolds composed of PEG-8-SH and PEG-8-Acr polymers grew for extended periods without requiring routine passaging or manipulation. The optimal growth of ESCs was achieved by using a concentration of 2.5 *w/v* % (dry weight of polymer per volume of culture medium) at a 1:1 molar ratio of PEG-8-SH and PEG-8-Acr. The results depicted in Figure 2 show steady cell growth up to 21 days as observed by light microscopy (Figure 2A–D). Cells grew clonally in a time-dependent manner. Visual observations were consistent with the results obtained by MTT assay. A significant and continuous increase in the proliferation of cells was observed during day 7 to 21. However, cells grew more rapidly between day 14 and 21 than between day 1 and 14 (Figure 2E), suggesting that the growth of ESCs required a longer acclimation period in 3-D self-assembling scaffolds compared to 2-D culture conditions. The viability of 3-D cultured ESCs was further validated by direct cell counts, as depicted in Figure 2F. The results showed that while cell proliferation significantly and consistently increased from day 1 to day 21, the number of dead cells remained low. 

To confirm whether H9 cells remained pluripotent during 3-D culture, the transcriptional and translational analysis of selected ESC-specific markers was performed using qRT-PCR and immunocytochemical analysis, respectively. The results indicated a successive increase in the expression of *OCT4*, *NANOG*, and *SOX2* throughout the duration of the 3-D culture, which increased 2.85, 2.23, and 3.69-fold, respectively in ESCs grown in 3-D scaffolds for 21 days as compared to cells grown under 2-D culture conditions (Figure 2G). The protein expression of these markers was also increased in cells cultured in the 3-D scaffolds (Figure 2H), which was consistent with the transcriptional upregulation.

### 3.2. Maintenance of Pluripotency in H9 Cells Grown under 3-D Culture Conditions

**Figure 3:**
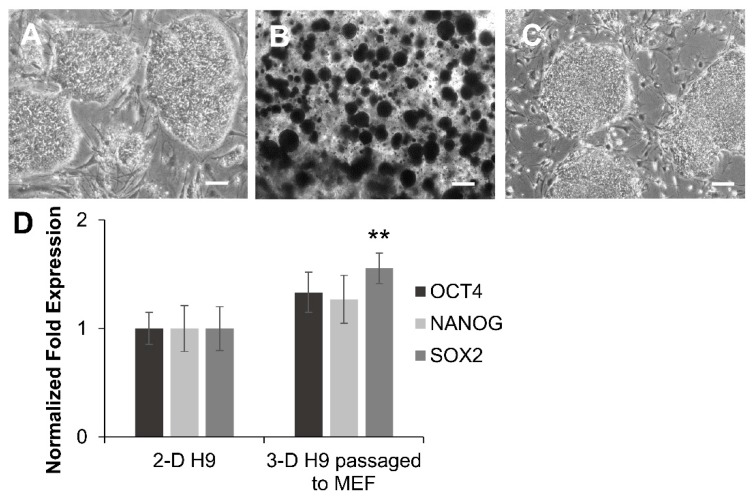
Self-renewal and pluripotency of ESCs were maintained following extended culture in 3-D scaffolds. (**A**–**C**) Cell morphology of ESCs (H9 cells) grown in 2-D cultures prior to encapsulation, in self-assembling scaffolds for 3-D culture for 21 days, and then subsequently subcultured back to 2-D culture conditions, respectively, as determined by light microscopy. All scale bars represent 100 μm. (**D**) Comparison of expression of *OCT4*, *NANOG*, and *SOX2* in ESCs grown in 2-D conditions and first in 3-D self-assembling scaffolds, and then passaged in 2-D culture conditions as determined by qRT-PCR. Results were expressed as the fold expression ± SE normalized to reference genes *HMBS*, *GAPDH,* and *β-ACTIN* (* *p* < 0.05 and ** *p* < 0.01).

To investigate if the 3-D grown H9 cells maintained their pluripotency after 21 days in 3-D culture, cells were subcultured under 2-D culture conditions and analyzed for cell morphology and the expression of pluripotent markers. The results showed that there were no morphological differences between cells that were passaged from 3-D to 2-D culture conditions and the initial ESCs used for encapsulation in the 3-D self-assembling scaffolds (Figure 3A–C). When H9 cells grown in 3-D self-assembling scaffolds for 21 days were subsequently subcultured under 2-D culture conditions, the expression of core pluripotent markers, *OCT4* and *NANOG*, was statistically similar to that of initial 2-D grown cells, while the expression of *SOX2* (1.55 fold) was slightly upregulated (Figure 3D). Apparently, repeated the subculturing of 3-D grown cells in 2-D culture conditions reverted the expression of these markers back to the normal level of expression. Despite the high expression of key pluripotent markers, it was prudent to further investigate the 3-D grown cells for the maintenance of their differentiation potential both in in vitro and in vivo.

### 3.3. Differentiation of H9 Cells Grown in 3-D Self-Assembling Scaffolds

**Figure 4:**
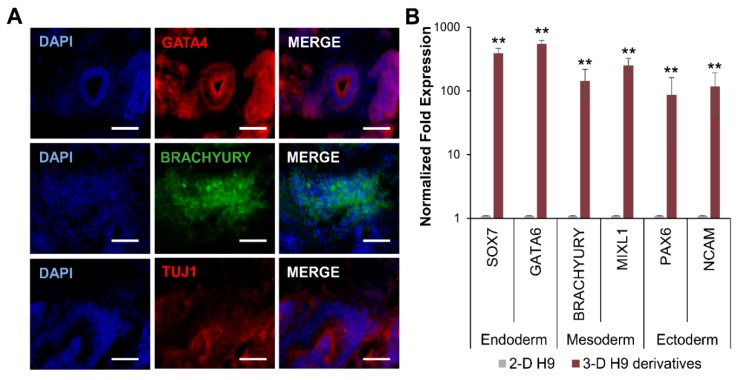
Differentiation potential of human ESCs grown in 3-D self-assembling scaffolds. Embryoid bodies (EBs) derived from 3-D grown ESCs (H9 cells) spontaneously differentiated into three germ layers and expressed specific proteins analyzed by immunocytochemistry. (**A**) Differentiated derivatives of 3-D grown ESCs expressed GATA4, BRACHYURY, and TUJ1 proteins representing the endoderm, mesoderm, and ectoderm, respectively, as shown by confocal images (20X). All scale bars represent 100 μm. (**B**) Differentiated derivatives of 3-D grown ESCs expressed germ layer-specific genes *SOX7* and *GATA6* (endoderm), *BRACHYURY* and *MIXL1* (mesoderm), and *PAX6* and *NCAM* (ectoderm) as determined by qRT-PCR. Results are expressed as the fold expression ± SE normalized to reference genes *HMBS*, *GAPDH,* and *β-ACTIN* (* *p* < 0.05 and ** *p* < 0.01).

The maintenance of the pluripotency of 3-D grown H9 cells was further investigated by the induction of differentiation into three germ layers following EB formation. The results presented in Figure 4 show that EBs from 3-D grown cells spontaneously differentiated into endoderm, mesoderm, and ectoderm germ layers, as evident by the protein expression of specific markers, GATA4, BRACHYURY, and TUJ1, respectively (Figure 4A). Transcriptional analysis also confirmed that differentiated derivatives of 3-D grown cells expressed markers of all three germ layers, including *SOX7* and *GATA6, BRACHYURY* and *MIXL1*, and *PAX6* and *NCAM,* representing the endoderm, mesoderm, and ectoderm, respectively (Figure 4B). 

**Figure 5:**
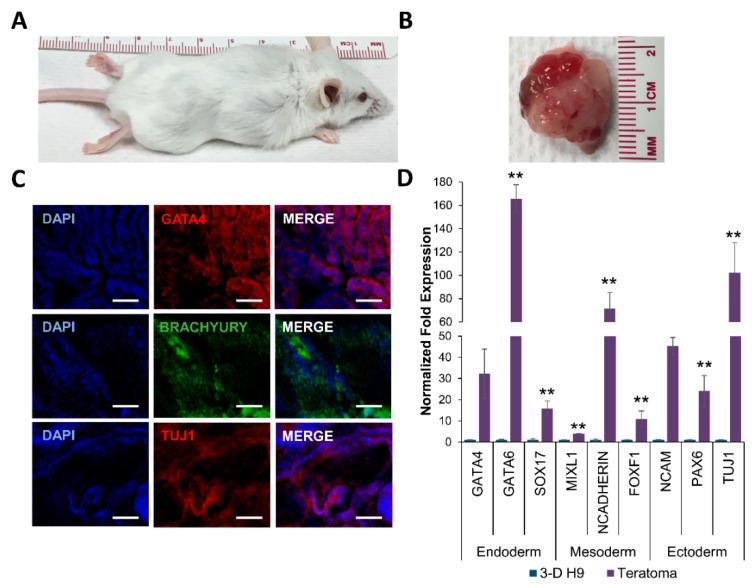
Teratoma formation by 3-D grown human ESCs in severe combined immunodeficient (SCID) Beige mice. (**A**) Tumor growth was observed in all mice (*n* = 3) injected with 3-D grown ESCs (H9 cells). (**B**) Explanted tumor at 4 weeks showed encapsulated, lobular, and well-circumscribed gross morphology consistent with teratoma growth. (**C**) Expression of GATA4, BRACHYURY, and TUJ1 proteins representing the endoderm, mesoderm, and ectoderm, respectively in excised teratomas, as shown by confocal images (20X). All scale bars represent 100 μm. (**D**) Expression of germ layer-specific genes, *SOX7* and *GATA6* (endoderm), *BRACHYURY* and *MIXL1* (mesoderm), and *PAX6* and *NCAM* (ectoderm) in excised teratomas, as determined by qRT-PCR. Results are expressed as the fold expression ± SE normalized to reference genes *HMBS*, *GAPDH,* and *β-ACTIN* (* *p* < 0.05 and ** *p* < 0.01).

Next, the pluripotency of 3-D grown H9 cells was validated in vivo by an analysis of teratoma formation. The results showed that the 3-D grown cells injected into SCID Beige mice formed teratomas (Figure 5). An analysis of teratoma tissue showed the expression of GATA4, BRACHYURY, and TUJ1 proteins, indicating that teratoma tissue had cells representing the endoderm, mesoderm, and ectoderm, respectively (Figure 5C). The transcriptional analysis of teratoma tissues also showed the expression of genes of all three germ layers: the endoderm (*SOX7*, and *SOX17*), mesoderm (*BRACHYURY*, and *MIXL1*), and ectoderm (*PAX6*, and *TUJ1*) (Figure 5D). Taken together, these results indicated that H9 cells grown in 3-D self-assembling scaffolds maintained their pluripotency and differentiation potential.

### 3.4. Expression of Naïve Pluripotent Markers in H9 Cells Grown under 3-D Culture Conditions and the Effect of YAP Inhibition

**Figure 6:**
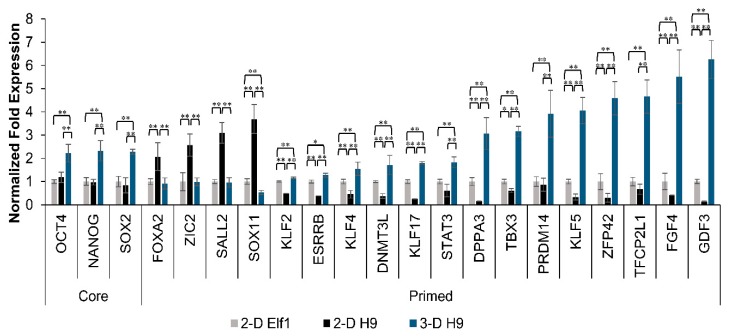
Effect of 3-D culture on the expression of naïve pluripotent markers in primed ESCs. (**A**) Expression of select core (*OCT4*, *NANOG*, and *SOX2*), primed (*FOXA2*, *ZIC2*, *SALL2*, and *SOX11*), and naïve (*KLF2*, *ESRRB*, *DNMT3L*, *KLF17*, *STAT3*, *DPPA3*, *TBX3*, *PRDM14*, *KLF5*, *ZFP42*, *TFCP2L1*, *FGF4*, and *GDF3*) pluripotent markers in ESCs cultured in 3-D scaffolds for 21 days and 2-D grown primed ESCs (H9 cells) and naïve ESCs (Elf1 cells, set to control) was analyzed by qRT-PCR. Results were expressed as the fold expression ± SE normalized to reference genes *HMBS*, *GAPDH*, and *β-ACTIN* (* *p* < 0.05 and ** *p* < 0.01).

Since a significant upregulation of core pluripotent markers, OCT4, NANOG, and SOX2, was observed in ESCs cultured in the 3-D scaffolds, we also assessed the expression of both primed and naïve pluripotent markers. A comparative transcriptional analysis of H9 cells grown in 3-D scaffolds and 2-D cultured H9 and Elf1 cells (representing primed and naïve ESC lines, respectively) is depicted in Figure 6. Expected primed pluripotent markers (*FOXA2*, *ZIC2*, *SALL2*, and *SOX11*) were expressed at higher levels in H9 than Elf1 cells cultured under 2-D conditions. Interestingly, the expression of primed pluripotent markers was significantly decreased in H9 cells grown in 3-D scaffolds to levels comparable to Elf1 cells grown under 2-D conditions. More strikingly, 3-D grown H9 cells expressed significantly higher levels of naïve markers (*KLF2*, *ESRRB*, *DNMT3L*, *KLF17*, *STAT3*, *DPPA3*, *TBX3*, *PRDM14*, *KLF5*, *ZFP42*, *TFCP2L1*, *FGF4*, and *GDF3*) in comparison to 2-D cultured Elf1 cells, suggesting that the 3-D scaffold microenvironment modulated gene expression. 

**Figure 7:**
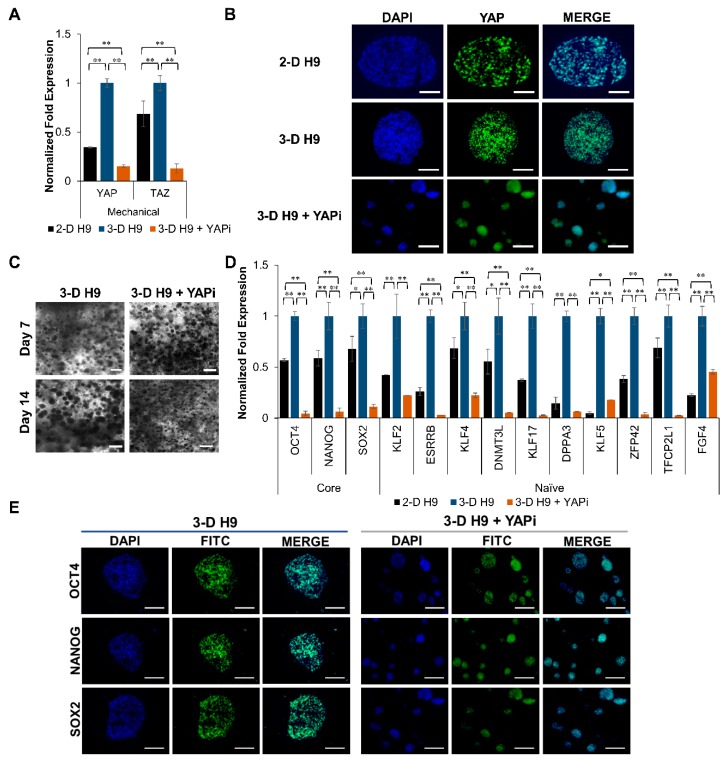
Effect of Yes-associated protein (YAP) inhibition (YAPi) on cell growth and expression of pluripotent markers in human ESCs cultured under 3-D conditions. ESCs (H9 cells) were grown under 3-D culture conditions for 14 days and incubated in the absence (control) or presence of a YAPi. (**A**) The effect of YAPi on the expression of mechanosensitive markers, *YAP* and *TAZ*, in H9 cells grown in 2-D and 3-D culture conditions as determined by qRT-PCR. (**B**) Merged confocal images (20X) showing YAP protein expression in 2-D and 3-D grown H9 cells. (**C**) Light micrographs depicting the cell morphology and colony size of 3-D grown H9 cells incubated in the presence or absence of YAPi. (**D**) Comparison of relative gene expression of core and naïve pluripotent markers in H9 cells grown under 2-D, 3-D, and 3-D + YAPi conditions as determined by qRT-PCR. Results were expressed as the fold expression ± SE normalized to reference genes *HMBS*, *GAPDH,* and *β-ACTIN* (* *p* < 0.05 and ** *p* < 0.01). (**E**) Merged confocal images (20X) of 3-D H9 cells with and without YAPi treatment for the protein expression of OCT4, NANOG, and SOX2. All scale bars represent 100 μm.

Many reports have stated that 3-D scaffolds induce differential gene expression due to mechanical and biological stimuli [10,38,39]. Since the overexpression of YAP has been shown to induce the naïve state of pluripotency in primed ESCs [40], we investigated the effect of YAP in 3-D cultured H9 cells using VP, which is a YAP inhibitor (YAPi). Our results in Figure 7A showed that mechanosensitive genes, *YAP* and *TAZ,* were upregulated in H9 cells grown in 3-D scaffolds in comparison to 2-D cultured cells. In contrast, *YAP* and *TAZ* expression significantly decreased when 3-D grown H9 cells were subjected to YAPi. A similar trend was observed at a translational level, where it appears that the immunofluorescence signal for YAP in 3-D grown H9 cells was brighter than in 2-D cultured cells. Moreover, an increased signal for YAP was observed in the cytoplasm of 3-D grown cells treated with YAPi (Figure 7B). 

The incubation of YAP also affected the clonal growth of H9 cells encapsulated in 3-D scaffolds, with YAPi-treated H9 cells exhibiting a significantly smaller colony size in comparison to untreated cells (Figure 7C). Interestingly, YAPi treatment also abrogated the upregulation of core (*OCT4*, *NANOG*, and *SOX2*), and naïve (*ESRRB*, *KLF4*, *DNMT3L*, *KLF17*, *DPPA3*, *KLF5*, *ZFP42*, *TFCP2L1*, and *FGF4*) pluripotent markers in 3-D cultured cells (Figure 7D). In addition, a concurrent decrease in the expression of core pluripotent proteins, OCT4, NANOG, and SOX2 was observed when compared with the untreated 3-D grown cells (Figure 7E). 

### 3.5. Mechanism of Regulation of Pluripotent Genes in H9 Cells Grown under 3-D Culture Conditions

**Figure 8:**
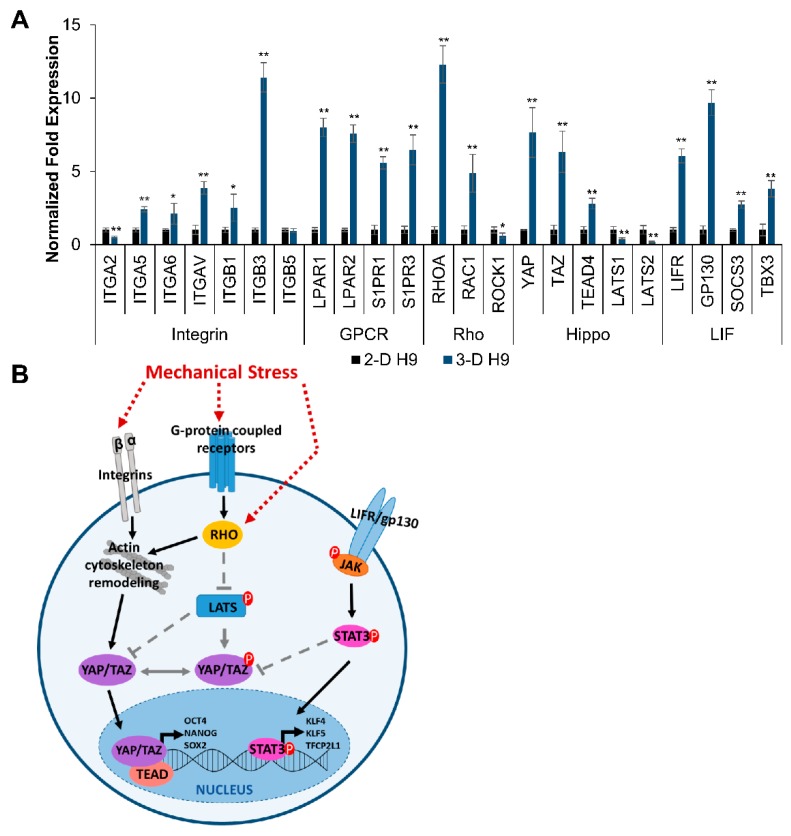
Molecular mechanism of upregulation of pluripotent markers in human ESCs grown in 3-D scaffolds. (**A**) The effect of 3-D culture of ESCs (H9 cells) on the expression of markers associated with integrin (*ITGA2*, *ITGA5*, *ITGA6*, *ITGAV*, *ITGB1*, *ITGB3*, and *ITGB5*) and G-coupled protein receptors (GCPRs; *LPAR1*, *LPAR2*, *S1PR1*, and *S1PR3*), Hippo (*YAP*, *TAZ*, *TEAD4*, *LATS1*, and *LATS2*), Rho (*RHOA*, *RAC1*, and *ROCK1*), and LIF (*LIFR*, *GP130*, *SOCS3*, and *TBX3*) signaling pathways as determined by qRT-PCR. Results were expressed as the fold expression ± SE normalized to reference genes *HMBS*, *GAPDH*, and *β-ACTIN* (* *p* < 0.05 and ** *p* < 0.01). (**B**) Proposed pathway involved in the induction of naïve pluripotency in primed human ESCs (H9 cells) encapsulated in 3-D self-assembling scaffolds.

To determine the basis for the upregulation of pluripotent markers observed in H9 cells grown in 3-D self-assembling scaffolds, we screened multiple signaling pathways with YAP-associated mechanotransduction using transcriptional analysis (Figure 8). The results depicted in Figure 8A show that 3-D cultured cells expressed higher levels of genes encoding integrin subunits, *ITGA5*, *ITGA6*, *ITGAV*, *ITGB1*, and *ITGB3* as well as G-coupled protein receptors (GCPRs), *LPAR1*, *LPAR2*, *S1PR1*, and *S1PR3.* In addition, these cells displayed the upregulation of *RHOA* and *RAC1* (Rho signaling), *YAP*, *TAZ*, and *TEAD4* (Hippo signaling), *LIFR*, *GP130*, *SOCS3*, and *TBX3* (LIF signaling). However, the expression of *ITGA2, ROCK1, LATS1*, and *LATS2,* which is associated with integrin, Rho, and Hippo signaling, respectively, decreased significantly, while *ITGB5* remained unchanged in 3-D cultured cells. 

These results further indicate that 3-D self-assembling scaffolds stimulated mechanosensitive signaling resulting in the upregulation of integrin receptors and GPCRs, thus promoting the activation of Rho signaling, which is associated with actin cytoskeleton remodeling; in turn, this led to the upregulation of mechanosensitive YAP/TAZ signaling. Activated YAP/TAZ act as transcription factors in the nucleus assisted by transcription co-factor, TEAD4, which binds to the DNA, to stimulate the expression of pluripotent genes. The simultaneous upregulation of LIF signaling also contributed to the upregulation of naïve pluripotent markers in H9 cells grown in 3-D self-assembling scaffolds, as proposed in Figure 8B.

## 4. Discussion

The expansion of human ESCs using traditional 2-D culture techniques is technically challenging and requires routine manipulation and passaging by dissecting colonies via enzymatic digestion or non-enzymatic methods [41,42,43]. These manipulations can result in poor viability, large batch-to-batch variation, and spontaneous differentiation. To address these problems, we hypothesized that 3-D culture may better mimic the in vivo environment from which ESCs are derived, which would improve both the long-term growth and maintenance of these cells.

Our study investigated the effect of the microenvironment in stemness by developing 3-D scaffolds made of two functionalized PEG polymers that self-assembled via a Michael addition reaction. When ESCs were included in the polymer mixtures, they were encapsulated upon self-assembly of the scaffolds. It has been previously reported that fully hydrated hydrogels mimic the 3-D native microenvironment, which allow nutrient diffusion and promote the growth of cells [44]. We have previously shown that soft 3-D scaffolds composed of Dex-SH and PEG-4-Acr self-assembling polymers were capable of supporting mouse ESC pluripotency for over 6 weeks [34]. Several other studies have also reported that mouse ESC self-renewal could be maintained on soft and low stiffness substrates in 2-D culture [45,46,47]. In addition, mechanically stiffer prefabricated scaffolds have been shown to promote the differentiation of mouse ESCs [48], while softer 3-D scaffolds supported the differentiation of human adult stem cells [49]. The culture of H9 cells in soft scaffolds did not support the viability of encapsulated cells, and growth was severely curtailed. This is consistent with previous studies, which showed that stiffer substrates promoted the maintenance of human ESCs [22,50,51]. Taken together, this led us to develop stiffer scaffolds made of functionalized PEG polymers.

To optimize scaffold polymerization as well as the encapsulation and growth of H9 cells, we tested several self-assembling polymers at various polymer concentrations in our preliminary studies (unpublished data). These studies showed that scaffolds made of PEG-8-SH and PEG-8-Acr prepared at 2.5% *w/v* improved the clonal growth of H9 cells in comparison to scaffolds with lower cross-linking densities and higher swelling ratios (i.e., Dex-SH/PEG-4-Acr and PEG-4-SH/PEG-4-Acr). These results were in line with other the reported studies performed using scaffolds made of multi-arm PEG functionalized with vinyl sulfone (VS) [52]. In these studies, H9 cells grew upon encapsulation in scaffolds made of VS functionalized 4-arm and 8-arm but not 3-arm PEG hydrogels [52]. However, this study was performed using clumps of H9 cells and not single cells for encapsulation. Whereas, in our study, PEG-8-SH/PEG-8-Acr scaffolds supported cell viability, allowing for even dispersal and clonal growth of H9 cells encapsulated as single cells. This has important implications because the growth of single cell inoculations and the generation of homologous populations of pluripotent cells is necessary for cell-based therapeutics [53]. Furthermore, the maintenance and growth of H9 cells was achieved using PEG-8-SH/PEG-8-Acr self-assembling scaffolds for long-term 3-D culture without passaging or manipulation. The pluripotency of the 3-D grown H9 cells was further demonstrated by their ability to differentiate into three germ layers and teratoma formation in vitro and in vivo, respectively. Additionally, core pluripotent markers, *OCT4*, *NANOG*, and *SOX2*, were upregulated during growth in the self-assembling scaffolds, showing significantly higher expression on day 21 of 3-D culture as compared to cells grown in 2-D culture. When 3-D grown H9 cells were passaged back to 2-D culture conditions, they exhibited undifferentiated morphology, and the expression of pluripotent markers decreased to levels similar to the 2-D cultured cells, suggesting that the encapsulated cells cultured under 3-D conditions were not altered.

Changes in the expression of core and naïve pluripotent markers in H9 cells cultured in 3-D self-assembling scaffolds can be attributed to multiple factors, including matrix dimensionality, stiffness, and/or bioinductive signaling [10]. While the incorporation of natural biomaterials has been shown to increase biological signaling, synthetic biomaterials lack biological activities minimizing batch-to-batch variation, but still allow for biophysical modifications, including pore size and mechanical stiffness [54]. Scaffolds composed of natural polymers including hyaluronic acid [55], chitosan, and alginate [56] have been shown to support human ESC self-renewal without a significant change in pluripotent marker expression. Whereas thermoresponsive synthetic hydrogels composed of PEG functionalized with poly-N-isopropylacrylamide allowed for the continuous 3-D culture of cells for 60 passages but only yielded cells 95% positive for *OCT4* [57]. In contrast, we observed the upregulation of not only *OCT4* but also *NANOG* and *SOX2* during the maintenance of human ESCs in 3-D scaffolds.

Previous studies with mouse ESCs reported that the differential upregulation of pluripotent markers in 3-D culture was dependent on scaffold composition and stiffness [34,58]. A recent study using single cell inoculation and the expansion of human ESCs in large-scale bioreactors resulted in the maintenance of *OCT4* levels but the upregulation of *SOX2* in 3-D grown ESC aggregates [59]. In another study, a 3-D culture of H9 clumps in VS functionalized PEG scaffolds resulted in upregulated gene expression of *SOX2* and *KLF4* but not *OCT4* and *NANOG* when compared to 2-D cultured cells [52]. Interestingly, we observed that primed ESCs, H9 cells, encapsulated in the 3-D self-assembling scaffolds exhibited a decreased expression of primed pluripotency markers, *FOXA2*, *ZIC2*, *SALL2*, and *SOX11*, which is characteristic of post-implantation epiblast cells [60,61], to levels comparable to naïve Elf1 cells. Furthermore, a 3-D culture of H9 cells resulted in an increase in the expression of naïve pluripotent markers, (*KLF2*, *ESRRB*, *DNMT3L*, *KLF17*, *STAT3*, *DPPA3*, *TBX3*, *PRDM14*, *KLF5*, *ZFP42*, *TFCP2L1*, *FGF4*, and *GDF3*), which was associated with naïve pre-implantation epiblast cells [7,60,61,62,63,64,65,66]. Recent studies have shown that traditionally derived primed human ESC lines can be reprogrammed to naïve pluripotency using the ectopic expression of select genes and specific media conditions containing inhibitor cocktails [67,68]. In one study, a transcriptomic comparison of primed and reprogrammed naïve human demonstrated the differential expression of certain genes such as *KLF2*, *KLF4*, *GDF3*, *SOCS3*, *STAT3,* and *TBX3* expressed at higher levels in naïve than primed cells [63]. However, the expression of core pluripotent markers *OCT4* and *NANOG* remained unchanged, while *SOX2* levels decreased upon reversion to naïve pluripotency [63]. Here, we observed an increase in core and naïve pluripotent markers during 3-D culture, which was presumably influenced by the 3-D scaffold microenvironment.

It has been well established that the transduction of biophysical signals, including substrate stiffness, cell–cell interactions, and mechanical forces can influence human ESC fate and self-renewal in 2-D culture conditions [39]. The interplay between external and internal mechanical stresses of cells and their ECM play important roles in tensional homeostasis of tissues in vivo [69]. However, the effect of 3-D culture on the regulation of the pluripotency of ESCs has not been fully explored.

Physical interactions with cells or the ECM can be transduced into biological signals and influence actin dynamics via mechanosensitive receptors, such as integrin receptors and GPCRs [70]. Evidence has shown that integrin heterodimers, α5β1, α6β1, and αvβ3, mediate interactions between ESCs and various ECM substrates [13,71,72,73]; they also play an important role in the maintenance of pluripotency [74], and matrix stiffness regulates integrin binding [75]. Whereas GPCRs, including lysophosphatidic acid receptors (LPARs) and sphingosine-1-phosphate receptors (S1PRs), play a role in the YAP signaling axis. In response to LPA and S1P ligand binding, the dephosphorylation of YAP and TAZ allows them to enter the nucleus to activate transcription [76]. Treatment with exogenous LPA has been shown to aid in the reversion of primed pluripotency to naïve pluripotency in ESCs due to the role that both YAP and LPA serve in suppressing differentiation associated with GSK3 inhibition [40]. However, the transduction of mechanical signals has also been shown to activate G-protein signaling independent of ligand binding [77]. We observed the upregulation of numerous integrin subunits as well as LPARs and S1PRs in 3-D grown H9 cells. This suggests that the 3-D scaffolds promoted mechanical signaling pathways in H9 cells; however, future studies are required to determine the mechanism by which these receptors are activated.

Mechanical signals such as substrate stiffness have been shown to lead to the activation of Rho signaling, which regulates actin cytoskeleton organization, leading to increased F-actin and nuclear localization of YAP and TAZ [78]. YAP is a mechanosensitive transcription factor in the Hippo signaling pathway, which plays a crucial role in cancer, regeneration, and the regulation of organ size [79]. In addition, Hippo signaling regulates the first cell fate specification to the trophoectoderm and the inner cell mass (ICM) of early blastocysts via mechanically sensitive pathways [80]. YAP is mostly retained in the cytoplasm in the ICM of early blastocysts, but becomes active during epiblast specification with a strong nuclear signal [64]. YAP and its transcription cofactor, TAZ, act as major downstream effectors in the Rho signaling pathway and have been shown to control the pluripotent state, allowing for the long-term survival and expansion of human ESCs in vitro [81].

Furthermore, cell culture on stiffer substrates has also been shown to increase the nuclear function of YAP/TAZ in human ESCs [82]. TAZ itself is required for the maintenance of self-renewal marker expression in ESCs, and the loss of TAZ leads to the inhibition of transforming growth factor beta (TGFβ) signaling and differentiation into a neuroectoderm lineage [83]. Likewise, a knockdown of YAP results in a loss of pluripotency in mouse ESCs, whereas the ectopic expression of YAP prevents differentiation [84].

In the nucleus, YAP mediates transcriptional enhanced associate domain (TEAD) transcription, and YAP/TAZ complexes associate with TEAD to regulate pluripotency by activating *OCT4* expression [85]. Pluripotency is determined by an autoregulatory core transcriptional circuitry comprised of *OCT4*, *NANOG*, and *SOX2,* which inhibits lineage specification genes [86]. The phosphorylation of YAP by LATS kinases prevents interaction with TEAD and results in cytoplasmic retention and the inactivation of YAP [87]. In contrast, YAP overexpression has been shown to promote the reversion of primed pluripotency into naïve pluripotency [40].

When 3-D grown H9 cells were cultured with a YAPi (VP), the upregulation of core and naïve pluripotent markers was abrogated. VP acts to inhibit the interaction of YAP and TEAD, disrupting downstream transcriptional activation, and sequestering YAP in the cytoplasm for inactivation [88]. Therefore, we postulate that mechanical forces in 3-D scaffolds stimulated the upregulation of mechanosensitive receptors, including integrins and GPCRs, leading to enhanced Rho signaling and higher levels of YAP/TAZ. In addition, the mechanical forces generated by the expansion of cells may also have contributed to the inhibition of phosphorylated LATS kinases [87], which in turn allowed YAP/TAZ to enter the nucleus, activating the transcription of pluripotent genes. This was concurrent with the observed increase in LIF signaling receptors and downstream naïve pluripotent target genes including *KLF4*, *KLF5,* and *TFCP2L1* [89]. While LIF/STAT3 signaling fails to maintain the self-renewal of primed human ESCs [90], naïve ESCs are dependent on LIF signaling [63]. Although these results are interesting and suggest a role of mechanical signaling in the regulation of cell fate in vitro, further analysis is needed to confirm the mechanism of upregulation of pluripotency markers and the genetic stability of ESCs grown in 3-D culture conditions.

Overall, the 3-D scaffolds made of PEG-8-SH/PEG-8-Acr support the clonal growth of primed ESCs as well as the enhanced expression of both core and naïve pluripotency markers, suggesting that the scaffold provided a permissive microenvironment for the induction of a naïve-like state of pluripotency. Our 3-D culture method is robust, simple, and less labor-intensive for the long-term amplification of homogenous populations of ESCs, which could promote their use in basic science and therapeutic applications.

## Figures and Tables

**Table 1:** List of human primer sequences used in qRT-PCR.

Gene	Primer Sequence		
	Forward (5′-3′)	Reverse (5′-3′)	Product Length
ACTIN	AATCTGGCACCACACCTTCTAC	ATAGCACAGCCTGGATAGCAAC	170
BRACHYURY	TGCTTCCCTGAGACCCAGTT	GATCACTTCTTTCCTTTGCATCAAG	121
DNMT3L	CTGCTCCATCTGCTGCTCC	ATCCACACACTCGAAGCAGT	85
DPPA3	AGACCAACAAACAAGGAGCCT	CCCATCCATTAGACACGCAGA	88
ESRRB	GACATTGCCTCTGGCTACCA	CTCCGTTTGGTGATCTCGCA	131
FGF4	CGTGGTGAGCATCTTCGGC	GTAGGACTCGTAGGCGTTGT	145
FOXA2	GGGAGCGGTGAAGATGGA	TCATGTTGCTCACGGAGGAGTA	89
FOXF1	AAGCCGCCCTATTCCTACATC	GCGCTTGGTGGGTGAACT	63
GAPDH	ACAACTTTGGTATCGTGGAAGG	GCCATCACGCCACAGTTTC	101
GATA4	TCCCTCTTCCCTCCTCAAAT	TCAGCGTGTAAAGGCATCTG	194
GATA6	CCCACAACACAACCTACAGC	GCGAGACTGACGCCTATGTA	131
GDF3	GTCTCCCGAGACTTATGCTACG	AGTAGAGGAGCTTCTGCAGGCA	136
GP130	GGAGTGAAGAAGCAAGTGGGA	AGGCAATGTCTTCCACACGA	128
HMBS	AGGAGTTCAGTGCCATCATCCT	CACAGCATACATGCATTCCTCA	104
ITGA2	TTGCGTGTGGACATCAGTCT	GCTGGTATTTGTCGGACATCT	158
ITGA5	GCCGATTCACATCGCTCTCAA	GTCTTCTCCACAGTCCAGCAA	139
ITGA6	CGAAACCAAGGTTCTGAGCCC	CTTGGATCTCCACTGAGGCAG	151
ITGAV	AGGAGAAGGTGCCTACGAAGC	GCACAGGAAAGTCTTGCTAAGG	105
ITGB1	GGATTCTCCAGAAGGTGGTTT	TGCCACCAAGTTTCCCATCT	143
ITGB3	CATGGATTCCAGCAATGTCCTC	TTGAGGCAGGTGGCATTGAAG	126
ITGB5	GCCTTTCTGTGAGTGCGACAA	CCGATGTAACCTGCATGGCAC	111
KLF17	TCAGGAAGGGACTGGTAGAA	GTACCCGCATATGTCGTCTAAG	206
KLF2	CCAAGAGTTCGCATCTGAAGG	CCGTGTGCTTTCGGTAGTG	132
KLF4	CGAACCCACACAGGTGAGAA	TACGGTAGTGCCTGGTCAGTTC	75
KLF5	ACCCTGGTTGCACAAAAGTT	CAGCCTTCCCAGGTACACTT	100
LATS1	CTCTGCACTGGCTTCAGATG	TCCGCTCTAATGGCTTCAGT	145
LATS2	ACATTCACTGGTGGGGACTC	GTGGGAGTAGGTGCCAAAAA	147
LIFR	CACCTTCCAAAATAGCGAGTATGG	ATGGTTCCGACCGAGACGAGTT	159
MIXL1	CCGAGTCCAGGATCCAGGTA	CTCTGACGCCGAGACTTGG	58
NANOG	AAAGAATCTTCACCTATGCC	GAAGGAAGAGGAGAGACAGT	110
N-CADHERIN	TGTTTGGCCTGGCGTTCTTT	AGGAGACAGAAACGAAGCCA	156
NCAM	AGGAGACAGAAACGAAGCCA	GGTGTTGGAAATGCTCTGGT	161
OCT4	CCCCTGGTGCCGTGAA	GCAAATTGCTCGAGTTCTTTCTG	97
PAX6	CTTTGCTTGGGAAATCCGAG	AGCCAGGTTGCGAAGAACTC	103
PRDM14	CCTTGTGTGGTATGGAGACTGC	CTTTCACATCTGTAGCCTTCTGC	126
RAC1	ATGTCCGTGCAAAGTGGTATC	CTCGGATCGCTTCGTCAAACA	249
RHOA	CATCCGGAAGAAACTGGT	TCCCACAAAGCCAACTC	168
ROCK1	GGTGGTCGGTTGGGGTATTTT	CGCCCTAACCTCACTTCCC	196
SALL2	GGCTTGCCTTATGGTATGTCCG	TGGCACTGAGTGCTGTTGTGGA	115
SOCS3	ATTCGGGACCAGCCCCC	AAACTTGCTGTGGGTGACCA	121
SOX11	CGACGACCTAATGTTCGACC	GACAGGGATAGGTTCCCCG	105
SOX17	CGCACGGAATTTGAACAGTA	GGATCAGGGACCTGTCACAC	182
SOX2	TTGCTGCCTCTTTAAGACTAGGA	CTGGGGCTCAAACTTCTCTC	75
SOX7	ACGCCGAGCTCAGCAAGAT	TCCACGTACGGCCTCTTCTG	73
STAT3	CTTTGAGACCGAGGTGTATCAC	GGTCAGCATGTTGTACCACAG	133
TAZ	GAGGGTGTATGGTGGAGATAAA	CCAACTGTAGCAAACAGGATTAG	86
TBX3	GGACACTGGAAATGGCCGAAG	GCTGCTTGTTCACTGGAGGAC	123
TBX3	CGGACATACTTGTTCCCCGA	GCAGGGTGAGCTGTTTTCTTTT	154
TEAD4	CCAAGCTCTGGATGTTGGAGTTC	GATGTCCACGGCTTCGAGGTA	161
TFCP2L1	TTTGTGGGACCCTGCGAAG	TGCTTAAACGTGTCAATCTGGA	129
TUJ1	GGCCAAGTTCTGGGAAGTCA	CGAGTCGCCCACGTAGTTG	70
YAP	GCTGCCACCAAGCTAGATAA	GTGCATGTGTCTCCTTAGATCC	101
ZFP42	CGCAATCGCTTGTCCTCAGA	GCTCTCAACGAACGCTTTCC	130
ZIC2	CGCTCCGAGAACCTCAAGAT	CCCTCAAACTCACACTGGAA	71

**Table 2:** List of primary and secondary antibodies used in immunocytochemical staining.

Antibody	Primary	Secondary
BRACHYURY	Rabbit Polyclonal	Anti-Rabbit Alexa Fluor 488
GATA4	Mouse Polyclonal	Anti-Mouse-Cy3
NANOG	Rabbit Polyclonal	Anti-Rabbit Alexa Fluor 488
OCT4	Rabbit Polyclonal	Anti-RabbitAlexa Fluor 488
SOX2	Rabbit polyclonal	Anti-Mouse Alexa Fluor 488
TUJ1	Mouse Polyclonal	Anti-Mouse-Cy3
YAP	Rabbit Polyclonal	Anti-Rabbit Alexa Fluor 488
